# Acute physiological responses and muscle recovery in females: a randomised controlled trial of muscle damaging exercise in hypoxia

**DOI:** 10.1186/s13102-024-00861-1

**Published:** 2024-03-22

**Authors:** Erich Hohenauer, G Bianchi, V Wellauer, W Taube, R Clijsen

**Affiliations:** 1 RESlab, University of Applied Sciences and Arts of Southern Switzerland, Weststrasse 8, CH-7302 Landquart, Switzerland; 2 International University of Applied Sciences THIM, Landquart, Switzerland; 3 University of Fribourg, Fribourg, Switzerland; 4 Department of Movement and Sport Sciences, Vrije Universiteit Brussel, Brussels, Belgium

**Keywords:** Hypoxia, Exercise, Training, Muscle damage, Recovery

## Abstract

**Background:**

Studies have investigated the effects of training under hypoxia (HYP) after several weeks in a male population. However, there is still a lack of knowledge on the acute hypoxic effects on physiology and muscle recovery in a female population.

**Methods:**

This randomized-controlled trial aimed to investigate the acute effects of muscle damaging exercise, performed in HYP and normoxia (CON), on physiological responses and recovery characteristics in healthy females. Key inclusion criteria were recreationally active female participants between the age of 18 to 35 years without any previous surgeries and injuries, whilst key exclusion criteria were acute pain situations, pregnancy, and medication intake. The females conducted a muscle-damaging protocol, comprising 5 × 20 drop-jumps, in either HYP (FiO_2_: 12%) or CON (FiO_2_: 21%). Physiological responses, including capillary oxygenation (SpO_2_), muscle oxygenation (SmO_2_), heart rate (HR), core- (Tcore) and skin- (Tskin) temperature were assessed at the end of each exercise set. Recovery characteristics were quantified by taking venous blood samples (serum creatine-kinase [CK], C-reactive protein [CRP] and blood sedimentation rate [BSR]), assessing muscle swelling of the quadriceps femoris muscle, maximum voluntary isometric contraction (MVIC) of the knee extensor muscles, countermovement jump (CMJ) performance and muscle soreness ratings (DOMS) at 24-, 48- and 72-hrs post-exercise.

**Results:**

SpO_2_ (HYP: 76.7 ± 3.8%, CON: 95.5 ± 1.7%, *p* < 0.001) and SmO_2_ (HYP: 60.0 ± 9.3, CON: 73.4 ± 5.8%, *p* = 0.03) values were lower (*p* < 0.05) in HYP compared to CON at the end of the exercise-protocol. No physiological differences between HYP and CON were observed for HR, Tcore, and Tskin (all *p* > 0.05). There were also no differences detected for any recovery variable (CK, CRP, BSR, MVIC, CMJ, and DOMS) during the 72-hrs follow-up period between HYP and CON (all *p* > 0.05).

**Conclusion:**

In conclusion, our results showed that muscle damaging exercise under HYP leads to reduced capillary and muscle oxygenation levels compared to normoxia with no difference in inflammatory response and muscle recovery during 72 h post-exercise.

**Trial registration:**

NCT04902924, May 26th 2021.

**Supplementary Information:**

The online version contains supplementary material available at 10.1186/s13102-024-00861-1.

## Introduction

Hypoxia can be defined as a reduction in the amount of O_2_ available to any cell, tissue, or organism [1]. A sudden reduction of O_2_ availability will instantly lead to specific physiological responses to optimize the supply of oxygen to metabolizing tissues [2].

One of the first experiences in high altitude exposures was carried out during the 18th century by balloonists [3]. These historic experiments were rather unpredictable, and the understanding of physiological hazards was limited. Later, research in the field of hypoxia focused on decision-making mistakes in pilots during the 1930s [4], the physiological responses during mountaineering [5] as well as physical performance during the Summer Olympic Games 1968 held in Mexico City, where altitude was a particularly pressing problem [6]. However, recent research in hypoxia has slightly shifted towards molecular mechanisms and consequences of hypoxia on cancer, since the discovery of the hypoxia-inducible factor-1(HIF-1) in the 1990s [7].

Altitude training for sport performance enhancement is an important topic in sport sciences but attracts relatively limited attention compared to the overall hypoxia research field [7, 8].

Traditionally, “live high - train high” or “live high - train low” training methods have been used in endurance and team sport athletes, to increase exercise capacity [9, 10]. Nowadays, with technological progress and accessibility, hypoxic training can be performed at sea level (normobaric hypoxia) and to increase muscular strength and hypertrophy [11]. It has been documented, that the adaptive effects of resistance training for hypertrophy and strength are highly dependent on applied loads and sets [12]. Exercise is a stressor with the ability to induce acute pro-inflammatory, systemic responses (e.g. elevations in C-reactive protein [CRP], creatine-kinase [CK], and others) [13, 14]. Interestingly, hypoxia has the potential to increase the production of reactive oxygen species and triggering pro-inflammatory response [15]. However, several factors, including mechanical, metabolic and neuronal factors are needed for muscular adaptions after exercise [16]. Especially unaccustomed, eccentric exercise can have a severe impact on the muscular system due to the mechanical impact and metabolic stress, associated with this type of exercise [17]. Plyometric exercises are established and valid methods to induce muscle-damage and inflammatory responses [18, 19]. From this perspective, plyometric exercise under hypoxia might have the ability to further stimulate pro-inflammatory cytokine release [20, 21], for inducing additional muscular adaptive processes.

Indeed, Nishimura et al. (2010) demonstrated, that moderate strength training under hypoxia (FiO_2_: 16%) resulted in improved muscle strength and hypertrophy compared to exercise under normoxia after a 6-week lasting training [22]. Inness et al. (2016) observed, that heavy resistance training under hypoxia (FiO2: 14.3%) over 7 weeks significantly increased absolute and relative strength compared to normoxic, heavy resistance training [23]. Similar results were detected in another study in more severe hypoxic condition (FiO_2_: 12.7%), where higher muscle hypertrophic changes were detected after an 8-week lasting moderate training protocol, compared to normoxic training [16]. Conversely, one study result could not detect significant differences compared to hypoxic conditions (FiO_2_: 12%) after a 4-week lasting moderate training intervention (6 × 25 knee extension exercise) on muscle strength and cross-sectional area [24]. These findings are in line with the study results from Ho et al. (2014), demonstrating that resistance training under hypoxia (FiO_2_: 15%) has no additive beneficial effect after a training period of 8 weeks on muscular performance [25]. Albeit these different findings in the literature, current evidence suggests potential promising applications of hypoxia for muscle hypertrophy and power training [11]. The lack of consensus in the literature might be attributed to different exercise protocols, training duration, exercise muscles, participants, as well as the hypoxic stimulus itself.

Although studies were carried out investigating the effects of exercise under hypoxia after different training programs over several weeks, there is a lack of studies that investigated acute physiological response and its impact on recovery. Additionally, females are still underrepresented in clinical trials, limiting biological understanding and contributing to health inequities and social justice [26]. One reason might be, that the acute response to hypoxia might differ between sexes. Examining the respiratory effects of hypoxia, Camacho-Cardenosa et al. (2022) observed that males exhibited an elevation in minute ventilation and a more pronounced initial decline in capillary oxygen saturation in comparison to females during a 7-hour exposure to moderate hypoxia (FiO_2_: 15%) [27]. Furthermore, Vento et al. (2022) identified markedly diminished capillary oxygenation levels and an increased occurrence of headache in females compared to males during acute hypoxia [28]. Despite the existence of studies that didn’t observe sex differences [29, 30], the inconsistence outcomes underpin the significance of investigating female responses to hypoxic stimuli. Therefore, our study aimed to compare the acute physiological effects after a single session of muscle damaging exercise, carried out under hypoxia (HYP) and normoxia (CON), and to evaluate the effects on subjective and objective recovery during a 72-hrs follow-up period.

## Methods

### Approach to the problem

The overall goal of this study was to compare the acute physiological effects after a single session of muscle damaging exercise, carried out under HYP and CON in healthy females, and to compare the effects on subjective and objective recovery characteristics during a 72-hrs period.

The muscle damaging exercise protocol comprised a plyometric task, where all participants conducted 5 × 20 drop-jumps with a 2-min break between the sets. The participants conducted this task either under hypoxia (FiO_2_: 12%) or normoxia (FiO_2_: 21%).

To investigate the acute physiological responses, capillary oxygenation, muscle oxygenation of the m. quadriceps femoris, heart rate, core- and skin temperature were assessed directly after each exercise set.

To observe the effects between exercising in hypoxia and normoxia on recovery characteristics, subjective (delayed onset of muscle soreness), and objective (pro-inflammatory cytokines, muscle swelling of the m. quadriceps femoris, countermovement-jump, and maximum voluntary isometric contraction of the knee extensors) parameters were assessed. These recovery characteristics were assessed at 24 h, 48 and 72 h after the muscle damaging exercise task.

### Participants

Female participants were eligible for the study, if they were healthy, between the age of 18 to 35 years, recreationally active (non-competitive physical endurance training, 2 to 3 times per week, 30 to 60 min per session) without previous injuries and surgeries. Participants were excluded from the study if they were smokers, pregnant, had acute injuries or painful situations, were exposed to altitude over 1000 m (including commercial flights) for at least one month, or were taking medication. They were instructed to maintain their normal daily habits, but 72 h before the experimental trial, participants were instructed to avoid strenuous exercise, alcohol, energy drinks, or other substances that could affect their physiological performance.

The participants were assigned either to the HYP group or the CON group using block randomisation by drawing lots. Two researchers (RC, EH) were responsible for the enrolment of participants, and screening the written inclusion and exclusion criteria from the questionnaire. One researcher (EH) was responsible for the random allocation sequence and assigning participants to the groups. Written informed consent was obtained from all study participants included in the study. Data collection took place between February 2022 and May 2022 in the laboratory (RESlab, University of Applied Sciences and Arts of Southern Switzerland, Landquart, Switzerland).

This study was approved by the local Ethical Committee of Zurich (2021 − 00546). The study is registered in the clinicaltrials.gov registry (NCT04902924; 26/05/2021) https://clinicaltrials.gov/ct2/show/NCT04902924. The CONSORT guidelines 2010 on reporting randomized trials were followed [31]. The S1 CONSORT 2010 flowchart and S2 CONSORT 2010 checklist can be found in the supplementary materials.

### Study design

This randomised controlled study was carried out over five experimental days using a parallel group design.

On day 1, participants filled out a screening questionnaire for eligibility (inclusion and exclusion criteria) and were familiarised with the experimental set-up (countermovement-jump on the jump mat and maximum voluntary isometric contraction on the ergometer chair).

On experimental day 2 (1 week after day 1), the anthropometric measurements were conducted, and the menstrual cycle data (using the calendar method) were recorded, to ensure that the hormonal levels in both groups are comparable. Then all baseline measurements were performed before assigning the participants randomly either to the HYP group or the CON group (block randomisation). After randomisation, the participants performed the muscle damaging exercise protocol, to induce moderate muscle damage (explained later). To evaluate the different physiological responses and the effects on muscle recovery between the conditions, the HYP group performed the exercise protocol at a PiO_2_ of 80 mmHg, while the CON group performed the same exercise task at a PiO_2_ of 140 mmHg.

The physiological parameters, capillary oxygenation (SpO_2_), muscle oxygenation (SmO_2_), heart rate, core temperature (Tcore) and skin temperature (Tskin) were measured at baseline, and immediately after each set (in total 5 sets) of the exercise protocol (ES1 to ES5), with the participant always in a standing position. Additionally, ratings of perceived exertion (RPE) and dyspnea (DYS) were also recorded at the end of each set of the protocol (ES1 to ES5).

**Fig. 1:**
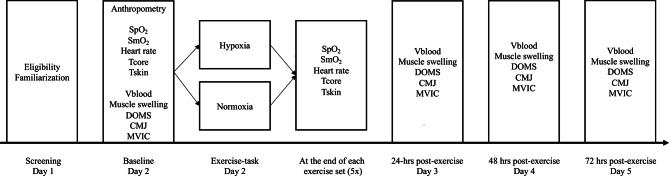
Schematic illustration of the experimental design

Markers of muscle recovery were assessed at baseline (day 2), 24- (day 3), 48- (day 4) and 72-hrs (day 5) after the muscle damaging exercise task, always in the following order: venous blood collection, anterior thigh muscle swelling, ratings of delayed onset of muscle soreness (DOMS), two-leg countermovement-jump (CMJ) performance and single-leg maximum voluntary isometric contraction (MVIC) of the right knee extensor muscles. A schematic representation of the test protocol is presented in Fig. 1.

### Muscle-damaging exercise protocol

A muscle damaging exercise protocol was chosen to induce muscle damage on the knee extensor muscles and comprised five sets (ES1 to ES5) of 20 drop-jumps from a 0.6 m box, as conducted previously [18]. According to the literature, this exercise task can be considered to induce moderate damage to the knee extensor muscles [32–34]. The participants had a 2-min break between each set, where the physiological parameters were assessed. The participants were allowed to sit down and rest after the parameters were collected. They were not allowed to drink water because of the potential influence of the ingested water on the Tcore data. The participants were verbally encouraged and instructed, if necessary (during the muscle-damaging exercise), to flex their knees at least 90° after the landing and to maintain their arms akimbo during the entire drop-jump.

### Hypoxic and normoxic environment

The participants performed the muscle damaging exercise task either under HYP or in the CON environment in the laboratory. In the HYP group, a custom-made (Mile High Training, Franklin NY, USA) tent (3.0 × 3.0 × 2.4 m) was used to create the normobaric hypoxic environment (FiO_2_: 12% [altitude of 4400 m], PB: 712 mmHg, PiO_2_: 80 mmHg). The tent was inflatable and an altitude generator (Everest summit II, Hypoxico, Bickenbach, Germany) was used to create an FiO_2_ of 12%. The hypoxic environment was controlled using an oxygen analyser (OxiQuant S, Envitec, Wismar, Germany). The participants entered the hypoxic tent and started the muscle-damaging exercise after breathing the hypoxic air for 2 min. During CON, the participants performed the same exercise protocol in the laboratory room (7.0 × 14.0 × 2.5 m) at a FiO_2_ of 21%, PB of 712 mmHg and a PiO_2_ of 140 mmHg, respectively.

### Physiological parameters during muscle damaging exercise

#### Capillary oxygen saturation

SpO_2_ from the left index finger was assessed using a pulse oximeter (Nonin 7500, Nonin medical b.v., Amsterdam, Netherlands). Pulse oximetry has been demonstrated to be a valid measurement tool until a desaturation value of 85% is reached in a hypoxic environment [35]. The values from the pulse oximeter (in %) were displayed and transmitted to a computer.

#### Muscle oxygen saturation

SmO_2_ was measured with the deep tissue oxygen monitor (moorVMS-NIRS, moor instruments, Millwey, UK), using probes that are placed on the skin [33, 36]. Each probe consists of a detector head that contains two identical photodiodes and an emitter head with two near-infrared LEDs, emitting light at approximately 750 and 850 nm. A standardised probe separation holder of 30 mm was used for reliability reasons. The probes were taped (Hypafix, BSN, Hamburg, Germany) on the muscle belly of the vastus lateralis of the right quadriceps femoris muscle, as previously described [36]. Oxygenated (OxyHb), deoxygenated (DeOxyHb) and total haemoglobin (TotHb) were collected in absolute values and expressed in arbitrary units. SmO_2_ was calculated (OxyHb / TotHb x 100) and expressed as a percentage value. The indirect assessment of muscle metabolism using near-infrared spectroscopy technology has been demonstrated to be a valid and reliable assessment tool [37].

### Heart rate

Heart rate was recorded using a polar watch (Polar, V800, Kempele, Finland) and a Bluetooth chest belt (Polar, H10, Kempele, Finland). The Polar V800 monitor has been demonstrated to be an accurate tool for the measurement of heart rate during exercise [38]. Heart rate values were analysed using absolute beats per minute (b^•^min^− 1^).

#### Core temperature

Tcore was recorded using the e-Celsius ingestible capsules and the e-Viewer (Body Cap, Caen, France). The capsule was ingested from the participants when entering the laboratory (day 2) with the instruction to swallow it immediately. Data were recorded every 30 s, stored within the capsule and transmitted to a computer at the end of the experiment before the participants left the laboratory. The ingestible temperature sensor has been demonstrated to represent a valid index of Tcore and to underestimate rectal temperature by 0.2 °C during exercise [39, 40]. Absolute values (°C) were used for the analyses.

#### Local skin temperature

Superficial Tskin of the anterior thigh was assessed using the conductive iButton (DS1922L) system (Maxim Integrated, San Jose, USA). The temperature logger was taped (K-active Tape Classic, Europe GmBh, Hoesbach, Germany) on the right anterior thigh, 2 cm above the probes for assessing SmO_2_. It has been demonstrated that the iButton system is a valid and reliable instrument for measuring skin temperature in humans [41]. Absolute values (°C) were used for the analyses.

### Subjective parameters during muscle damaging exercise

#### Ratings of perceived exertion

The participant’s RPE were assessed using a vertical BORG scale, ranging from 6 “no exertion” to 20 “maximum exertion” [42], which were used for the analyses. Before the experimental procedure, the participants were familiarized with the RPEs scale. At baseline and the end of each exercise set, the participants were asked to rate their physical exertion level by telling one number.

#### Ratings of dyspnea

Acute DYS was assessed using the modified BORG scale for dyspnea, ranging from 0 “no dyspnea” to 10 “maximum dyspnea” [43]. The modified BORG scale for dyspnea has been demonstrated to be an instrument for clinical practice to measure dyspnea at exercise, with a moderate specificity [44]. Dyspnea status was assessed at baseline and the end of each exercise set.

### Recovery parameters after muscle damaging exercise

#### Venous blood sample

A venepuncture from the antecubital fossa was performed to collect blood from the participants. For the blood sedimentation rate (BSR), a venous blood sample was collected in 5.0 ml EDTA tubes (BD Seditainer, Plymouth, UK). Samples were placed into the sedimentation measurement stand (BD Seditainer, Plymouth, UK). After the sample rested for one hour in a vertical position, the BSR was reported in mm/hr, according to the Westergren method [45]. For the assessment of CRP and CK, an additional venous blood sample was collected in 8.5 ml tubes (BD Vacutainer, Plymouth, UK), centrifuged (3000 g for 10 min; Hettich, EBA 20, Baech, Switzerland) and analysed using the turbidimetric method (CRP) [46] and using an automated ultraviolet method (Roche, Basel, Switzerland). The venous blood samples were analysed using normalised values to baseline.

#### Muscle swelling

Muscle swelling of the right quadriceps femoris muscle was evaluated using an ultrasound system (MyLabClass C, Esaote, Genoa, Italy) in B-mode. By using the minimal pressure technique, the distance between the femoral bone and the outer layer of the quadriceps muscle (excluding the overlying adipose tissue) was used to evaluate muscle swelling. For reliable measurements over the experimental days, the measurement position for the ultrasound probe was marked, at 60% of the distance between the greater trochanter and the lateral epicondyle, 3 cm lateral to the midline [18, 33]. Normalised values to baseline were used for the analyses.

#### Delayed onset of muscle soreness

Subjective anterior thigh soreness was rated using a horizontal VAS, ranging from 0 to 100 mm [47]. Participants were instructed to rate their DOMS status during a 3-second lasting 90° squatted position. The far left of the scale (0 mm) indicated no soreness at all, and the far right of the scale (100 mm) indicated severe muscle soreness. Absolute values (mm) were used for the analyses)

#### Countermovement jump

The maximum CMJ performance was assessed on a jump mat (Just jump, Probotics Inc, Huntsville, USA). The participants were instructed to stand on the mat, place their hands on their hips, and perform a maximum CMJ [47]. A total of three attempts were performed in a row and the highest values were used for the analyses. The participants were not verbally encouraged and remained blinded to the CMJ values throughout the experiment. Values were normalised to baseline for the analyses.

#### Maximum voluntary isometric contraction

The MVIC (measured in kg) of the right knee extensor muscles was assessed in a seated position on an ergometer chair (EROS-1, Landquart, Switzerland). The participant’s right leg was positioned with a knee angle of 120° and a hip angle of 100°, to obtain reliable results [33]. After positioning, the participants were instructed to perform a maximum isometric contraction for the duration of three seconds, without verbal encouragement. A total of three sets were performed with a one-minute break in between and the maximum value was taken for the analyses. The participants were blinded to the MVIC values throughout the experiment. MVIC values were normalised to baseline for the analyses.

### Sample size

Using data from a study employing a similar methodological design [18] the sample size was determined using G*Power (version 3.1.9.3) [48]. The following design specifications were considered: α = 0.05; 1-β = 0.8; f = 0.4; statistical test = Repeated Measures ANOVA with within-between interaction. The total sample size estimated according to these specifications was *n* = 12 participants.

### Statistical analyses

The statistical analyses were performed using SPSS statistics V. 28 (IBM Corp., Armonk, USA), with the significance level set at *p* < 0.05. Descriptive results are reported as means ± standard deviations (SD). The assumption of normality was assessed using the Shapiro-Wilk test. Mixed design repeated measures ANOVAs were used to assess the main effects of time (physiology and subjective variables: baseline, after exercise set 1, set 2, set 3, set 4, and set 5; recovery variables: baseline, 24-hrs, 48-hrs, 72-hrs) as within factor, condition (HYP, CON) as between factor, and to assess interaction effects (time*condition). Post-hoc analyses were performed using Tukey’s HSD test where appropriate. The effect sizes were expressed as partial eta-squared (η^2^_partial_) with values of 0.1 to 0.29, 0.30 to 0.49, and > 0.5 considered as small, medium, and large, respectively [49].

## Results

### General characteristics of the participants

Twenty female participants, regularly involved in moderate physical endurance activity, volunteered for this study. The results of all (*n* = 20) included participants were analysed in this study (HYP *n* = 10, CON *n* = 10) There were no significant differences between groups regarding anthropometric characteristics and menstrual cycle phase (all *p* > 0.05, Table 1).

**Table 1:** Characteristics of the *n* = 20 female participants

Parameters	HYP (*n* = 10)	CON (*n* = 10)	p-value
Age (years)	23.8 ± 3.1	23.0 ± 1.6	0.48
Height (cm)	164.9 ± 6.7	168.8 ± 5.2	0.17
Body mass (kg)	58.7 ± 6.6	62.2 ± 7.2	0.27
BSA (m^2^)	1.6 ± 0.1	1.7 ± 0.1	0.17
BSA: mass (m^2^.kg^− 1^)	0.02 ± 0.001	0.02 ± 0.001	0.54
Body fat %	17.2 ± 5.6	20.6 ± 7.5	0.06
BMI	21.5 ± 2.2	21.8 ± 2.4	0.81
Menstruation ph. (%)	20	10	1.0
Proliferative ph. (%)	50	40	1.0
Secretory ph. (%)	30	50	0.72

Note: BSA = body surface area, BMI = body mass index, ph = phase, values are means ± SD

### Physiological parameters during the muscle damaging exercise task

#### Capillary oxygen saturation

**Fig. 2:**
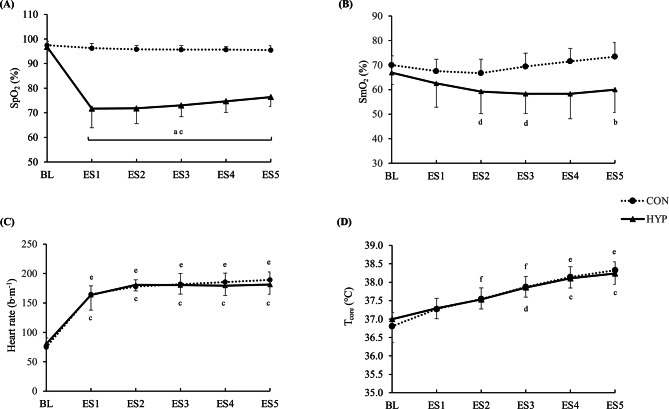
Physiological responses during the exercise task. Results of (**A**) capillary oxygen saturation (SpO_2_), (**B**) muscle oxygen saturation of the right vastus lateralis of the quadriceps femoris muscles (SmO_2_), (**C**) heart rate, and (**D**) core temperature (T_core_) in function of time in the HYP and CON groups. BL baseline, ES exercise-set, ^a^*P* < 0.001 HYP vs. CON, ^b^*P* < 0.05 HYP vs. CON, ^c^*P* < 0.001 HYP compared with baseline, ^d^*P* < 0.05 HYP compared with baseline, ^e^*P* < 0.001 CON compared with baseline, ^f^*P* < 0.05 CON compared with baseline

SpO_2_ values were only significantly lower in HYP compared to CON during the exercise task compared to baseline, with significant differences between the two conditions (Fig. 2A).

A significant time (*p* < 0.001, η^2^_partial_ = 0.80), condition (*p* < 0.001, η^2^_partial_ = 0.91) and interaction (*p* < 0.001, η^2^_partial_ = 0.76) was found for SpO_2_.

SpO_2_ was lower in HYP compared to CON throughout (all *p* < 0.001) the exercise task (ES1: 71.7 ± 7.7% vs. 96.3 ± 1.9%; ES2: 71.8 ± 6.2% vs. 95.8 ± 1.6%; ES3: 73.0 ± 4.5% vs. 95.7 ± 1.6%; ES4: 74.7 ± 4.5% vs. 95.7 ± 1.1%; ES5: 76.4 ± 3.8% vs. 95.5 ± 1.7%).

Compared to baseline values (96.8 ± 1.6%) in HYP, values were lower (all *p* < 0.001) within this condition between ES1 and ES5. No within-condition differences (all *p* > 0.05) were detected in CON between baseline (97.5 ± 1.5%) and ES1 to ES5.

#### Muscle oxygenation

SmO_2_ decreased significantly in HYP during the exercise task compared to baseline, but not in CON (Fig. 2B).

For SmO_2_, a main effect of time (*p* = 0.003, η^2^_partial_ = 0.18), condition (*p* = 0.003, η^2^_partial_ = 0.38) and an interaction effect (*p* < 0.001, η^2^_partial_ = 0.20) was detected.

A trend for reduced SmO_2_ values in HYP compared to CON was detected at ES3 (58.3 ± 8.0% vs. 69.4 ± 5.4%, *p* = 0.06) and ES4 (58.3 ± 10.2% vs. 71.5 ± 5.3%, *p* = 0.06), which reached significance at ES5 (60.0 ± 9.3% vs. 73.4 ± 5.8%, *p* = 0.03).

In HYP, values decreased significantly compared to baseline (66.9 ± 4.8%) at ES2 (59.2 ± 9.0, *p* = 0.02), ES3 (58.3 ± 8.0%, *p* = 0.01), reaching borderline to significance at ES4 (58.3 ± 10.2%, *p* = 0.05).

In CON, no significant differences within the condition were detected between baseline (70.0 ± 3.7%) and ES1– ES5 (all *p* > 0.05).

#### Heart rate

Heart rate was significantly elevated in both HYP and CON compared to baseline, with no differences between conditions (Fig. 2C).

For heart rate, only a main effect of time (*p* < 0.001, η^2^_partial_ = 0.93), with no condition (*p* = 0.79, η^2^_partial_ = 0.01) and interaction effect (*p* = 0.41, η^2^_partial_ = 0.18) was detected. Albeit no significant differences between conditions were observed at specific time points (baseline, ES1 to ES2), increased heart rates were observed within groups (HYP and CON). In HYP, heart rate was higher compared to baseline (80.4 ± 8.6 bpm) ranging from ES1 (163.7 ± 25.8, *p* < 0.001) to ES5 (181.3 ± 16.5 bpm, *p* < 0.001). Also in CON, values were higher compared to baseline (75.0 ± 15.5 bpm) from ES1 (164.5 ± 14.5 bpm, *p* < 0.001) to ES5 (189.2 ± 13.5, *p* < 0.001).

#### Core temperature

Core temperature was significantly higher in HYP and CON during the exercise task compared to baseline, with no between-condition differences (Fig. 2D).

A significant effect of time (*p* < 0.001, η^2^_partial_ = 0.85), with no condition (*p* = 0.92, η^2^_partial_ = 0.01) and interaction (*p* = 0.92, η^2^_partial_ = 0.01) was observed. There were no differences detected between HYP and CON at any time point (all *p* > 0.05).

However, within the condition, core temperature was higher in HYP compared to baseline (37.0 ± 0.6 °C) at ES3 (37.9 ± 0.3, *p* = 0.003), ES4 (38.1 ± 0.3 °C, *p* < 0.001), and ES5 (38.2 ± 0.3 °C, *p* < 0.001). In CON, skin temperature was different to baseline (36.8 ± 0.4 °C) at ES2 (37.5 ± 0.2 °C, *p* = 0.02), ES3 (37.9 ± 0.3 °C, *p* = 0.004), ES4 (38.1 ± 0.3 °C, *p* < 0.001), and ES5 (38.3 ± 0.2 °C, *p* < 0.001).

#### Skin temperature

There were no detectable changes in skin temperature in HYP and CON during the exercise task compared to baseline (Table 2).

No main effects for time (*p* = 0.81, η^2^_partial_ = 0.02), condition (*p* = 0.79, η^2^_partial_ = 0.01), and interaction (*p* = 0.90, η^2^_partial_ = 0.01) were found for skin temperature. There were no differences observed between the HYP and CON conditions at any time point (all *p* > 0.05). Also, within the condition, no differences to baseline were observed for HYP (30.4 ± 1.4 °C) and CON (30.2 ± 2.8 °C).

**Table 2:** Physiological characteristics of skin temperature and subjective ratings during the muscle damaging exercise task (means ± SD)

	BL	ES1	ES2	ES3	ES4	ES5	p-value within
**Tskin(°C)** HYP CON	30.4 ± 1.4 30.2 ± 2.8	30.6 ± 1.4 30.6 ± 1.6	30.5 ± 1.3 30.5 ± 1.3	30.6 ± 1.4 30.5 ± 1.4	30.8 ± 1.5 30.4 ± 1.4	30.9 ± 1.6 30.4 ± 1.8	All > 0.05 All > 0.05
**RPE** **(BORG 6to20)** HYP CON	6.4 ± 0.8 6.5 ± 1.0	14.2 ± 1.0 12.8 ± 1.2	14.8 ± 1.4 13.9 ± 1.2	15.4 ± 0.8 14.5 ± 1.6	16.3 ± 1.5 15.6 ± 1.8	16.7 ± 1.3 16.1 ± 2.2	All < 0.001 All < 0.001
**DYS ** **(BORG 0to10)** HYP CON	0.2 ± 0.3 0.0 ± 0.0	3.1 ± 0.7 3.0 ± 1.2	4.0 ± 1.1 3.6 ± 1.2	4.9 ± 1.3 4.1 ± 1.4	5.4 ± 1.8 5.0 ± 2.6	5.9 ± 1.8 5.6 ± 2.2	All < 0.001 All < 0.001

Legend: Tskin local skin temperature, RPE ratings of perceived exertion, DYS dyspnea, HYP hypoxia, CON normoxia

### Perceptual parameters during the muscle damaging exercise task

#### Ratings of perceived exertion

RPE values increased in both conditions during the exercise task compared to baseline, with no between-group differences (Table 2).

There was only an effect of time (*p* < 0.001, η^2^_partial_ = 0.93) detected for RPE with no condition (*p* = 0.13, η^2^_partial_ = 0.11) and interaction effect (*p* = 0.29, η^2^_partial_ = 0.06).

In both conditions, RPE was higher compared to the baseline throughout the exercise task (*p* < 0.001 for HYP and CON).

#### Dyspnea

Dyspnea was elevated during the exercise task in both conditions, with no differences between HYP and CON (Table 2). The analyses revealed a main effect of time (*p* < 0.001, η^2^_partial_ = 0.82) for dyspnea, but no condition (*p* = 0.49, η^2^_partial_ = 0.02) and interaction effect (*p* = 0.91, η^2^_partial_ = 0.01).

In both conditions, DYS was higher compared to the baseline throughout the exercise task (*p* < 0.001 for HYP and CON).

### Recovery parameters after the muscle damaging exercise task

#### Inflammatory blood markers

All inflammatory blood markers (CK, CRP, and BSR) increased during the recovery period compared to baseline, with no differences between conditions (Table 3).

For CK, a significant effect of time (*p* < 0.001, η^2^_partial_ = 0.29), with no condition (*p* = 0.75, η^2^_partial_ = 0.01) and interaction effect (*p* = 0.48, η^2^_partial_ = 0.04) was detected, Table 3. No between (*p* > 0.05) and within (*p* > 0.05) conditions differences were detected at any specific time point. After 24-hrs, the relative values peaked in HYP (538.5 ± 655%) and CON (372.4 ± 235.6) with no difference to baseline.

For CRP, a significant time (*p* < 0.001, η^2^_partial_ = 0.29), but no condition (*p* = 0.41, η^2^_partial_ = 0.03) and interaction effect (*p* = 0.71, η^2^_partial_ = 0.02) was detected, Table 3.

Albeit no differences were detected between (HYP vs. CON) or within groups, the CRP values peaked in both groups after 24-hrs compared to baseline (HYP: 127.0 ± 46.1%; CON:119.3 ± 26.3%).

For BSR, the analyses showed a main effect of time (*p* = 0.001, η^2^_partial_ = 0.25) but no condition (*p* = 0.74, η^2^_partial_ = 0.01) and interaction effect (*p* = 0.54, η^2^_partial_ = 0.03), Table 3.

No significant differences between HYP and CON were detected throughout the recovery period (24-hrs, 48-hrs, 72-hrs; all *p* > 0.05). No differences were detected from baseline values within conditions (HYP and CON). Albeit no change from baseline was detected in both groups, BSR peaked after 48-hrs in HYP (172.1 ± 105.8%) and CON (149.8 ± 55.3%).

**Table 3:** Recovery characteristics of inflammatory blood marker after the muscle damaging exercise task. (Normalised to baseline ± SD)

	BL	24-hrs	48-hrs	72-hrs	p-value within
**CK %**					
HYP CON	100 ± 0 100 ± 0	538.5 ± 655.3 372.4 ± 235.6	268.4 ± 266.3 236.3 ± 159.7	187.7 ± 149.3 256.1 ± 402.3	All > 0.05 All > 0.05
**CRP %**					
HYP CON	100.0 ± 0 100.0 ± 0	127.0 ± 46.1 119.3 ± 26.3	117.0 ± 27.0 104.8 ± 12.8	105.9 ± 12.4 100.8 ± 10.0	All > 0.05 All > 0.05
**BSR %**					
HYP CON	100.0 ± 0 100.0 ± 0	128.3 ± 50.1 112.6 ± 39.1	172.1 ± 105.8 149.8 ± 55.3	126.0 ± 54.0 142.4 ± 43.1	All > 0.05 All > 0.05

Legend: CK creatine kinase, CRP C-reactive protein, BSR blood sedimentation rate, % normalised to baseline, HYP hypoxia, CON normoxia

#### Muscle swelling

**Fig. 3:**
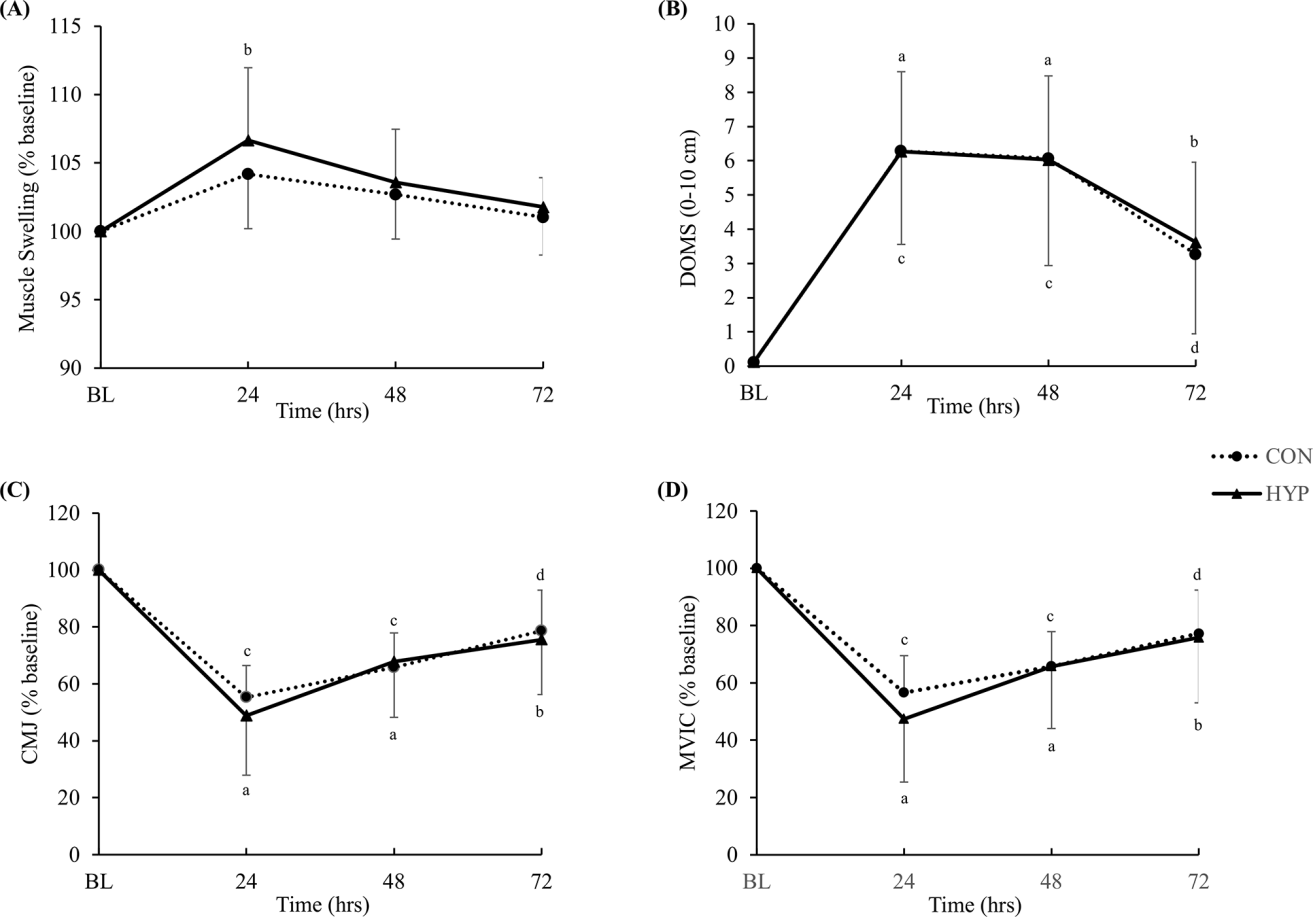
Subjective and objective recovery characteristics after the exercise task. Results of (**A**) muscle swelling, (**B**) delayed onset of muscle soreness (DOMS), (**C**) countermovement jump (CMJ), and (**D**) maximum voluntary isometric contraction (MVIC) in function of time in the HYP and CON groups. Values for A, C, and D are normalised to baseline (% mean ± SD) with respect to their initial values. BL baseline, ^a^*P* < 0.001 HYP compared with baseline, ^b^*P* < 0.05 HYP compared with baseline, ^c^*P* < 0.001 CON compared with baseline, ^d^*P* < 0.05 CON compared with baseline

In HYP, muscle swelling was increased after 24 h compared to baseline, but there were no differences between conditions (Fig. 3A).

For muscle swelling of the right quadriceps femoris muscle, a main effect of time was detected (*p* < 0.001, η^2^_partial_ = 0.44) with no condition (*p* = 0.29, η^2^_partial_ = 0.06) and interaction effect (*p* = 0.56, η^2^_partial_ = 0.03). In CON, there was no significant difference compared to baseline values throughout the 72-hrs lasting recovery period (*p* > 0.05 for all time points). However, in HYP, muscle swelling increased significantly (*p* = 0.006) after 24-hrs (106.7 ± 5.3%) and demonstrated a statistical trend (*p* = 0.07) to remain increased after 48-hrs (103.6 ± 3.9%) compared to baseline.

#### Delayed onset of muscle soreness

DOMS values increased in both conditions compared to baseline, with no differences between conditions (Fig. 3B). For DOMS, a significant effect for time (*p* < 0.001, η^2^_partial_ = 0.79) but not for condition (*p* = 0.92, η^2^_partial_ = 0.01) and interaction effect (*p* = 0.97, η^2^_partial_ = 0.01) were detected.

There were no significant differences between conditions at any time point. Albeit no statistical between-condition differences, significant within-condition differences were detected for both HYP and CON. In HYP, DOMS was higher compared to baseline (0.0 ± 0.0 cm) after 24-hrs (6.2 ± 2.3 cm, *p* < 0.001), after 48-hrs (6.0 ± 2.4 cm, *p* < 0.001) and after 72-hrs (3.6 ± 2.3 cm, *p* = 0.003). Also in CON, DOMS was higher compared to baseline (0.0 ± 0.0 cm) after 24-hrs (6.2 ± 2.7 cm, *p* < 0.001), after 48-hrs (6.0 ± 3.1 cm, *p* < 0.001), and after 72-hrs (3.2 ± 2.3 cm, *p* = 0.008).

#### Countermovement jump

In both conditions, significant reductions in CMJ-performance were detectable, with no differences between conditions (Fig. 3C).

There was an effect of time (*p* < 0.001, η^2^_partial_ = 0.80) but no condition (*p* = 0.71, η^2^_partial_ = 0.01) and interaction effect (*p* = 0.60, η^2^_partial_ = 0.03) detected for CMJ. Compared to baseline, values were lower in HYP after 24-hrs (48.8 ± 20.9%, *p* < 0.001), 48-hrs (67.8 ± 19.6%, *p* < 0.001) and 72-hrs (75.5 ± 19.3%, *p* = 0.005). Also in CON, values were lower compared to baseline after 24-hrs (55.3 ± 11.2%, *p* < 0.001), 48 h (65.8 ± 12.1%, *p* < 0.001) and remained lower after 72-hrs (78.6 ± 14.4%, *p* = 0.01).

#### Maximum voluntary isometric contraction

MVIC decreased compared to baseline in HYP and CON, with no between-condition differences (Fig. 3D). For MVIC, a main effect of time (*p* = 0.001, η^2^_partial_ = 0.76) but no effect for condition (*p* = 0.63, η^2^_partial_ = 0.01) and interaction (*p* = 0.54, η^2^_partial_ = 0.03) was detected.

In HYP, compared to baseline, MVIC values decreased to 47.4 ± 22.0% (*p* < 0.001) after 24-hrs, to 65.8 ± 21.7% (*p* < 0.001) after 48-hrs and to 75.8 ± 22.8% (*p* = 0.01) after 72-hrs. In CON, MVIC values also decreased after 24-hrs to 56.6 ± 12.9% (*p* < 0.001), to 65.8 ± 12.1% (*p* < 0.001) after 48-hrs and to 77.2 ± 15.2% (*p* = 0.02) after 72-hrs.

## Discussion

This randomized controlled trial aimed to investigate the acute effects of muscle damage under hypoxia and normoxia on physiological differences and the effects on recovery during 72 h post-exercise, in healthy females.

The main findings of this study are that SpO_2_ and SmO_2_ remained lower during muscle damaging exercise in hypoxia compared to exercise under normoxia. No differences between hypoxia and normoxia were detected in subjective (DOMS) and objective (inflammatory blood markers, muscle swelling, CMJ, and MVIC) recovery characteristics during a 72-hrs follow-up period.

From a practical perspective, it would be acceptable to assume, that exercise under hypoxia would lead to higher inflammatory responses compared to exercise under normoxia. This hypothesis could arise from the observation, that exercise induced,- muscle damaged tissue, and the following inflammatory responses are characterized by O_2_ and nutrient deprivation [50], leading to an enhanced performance adaption. As a response, immune cells, including neutrophils, monocytes and eosinophils, infiltrate these hypoxic tissues to generate an appropriate response to tissue damage [51–53]. Interestingly, a direct performance output benefit, arising from exercise under hypoxia, is rarely observed in the literature [54]. Similarly, high-intensity exercise under hypoxia did not induce a more severe inflammatory response than the same exercise load, performed under normoxia [55, 56]. However, compared to normoxic training, hypoxic training has the potential to elicit specific molecular adaptations [57, 58], but these are not necessarily associated with improved physical performance [54]. On the other side, it has been postulated that both hypoxia and inflammation-induced activation of immune cells lead to the release of the master regulator of oxygen homeostasis, namely HIF-1 [59]. Although not measured in our study, the release of HIF-1α is different in exercise under normoxia compared to hypoxia [57]. Although the precise role of HIF-1α as a response to increased oxygen demand is not fully elucidated [60], it is known that mammalian skeletal muscle tissue has a high adaptive capacity to change the metabolic characteristics, as a result of deprived O_2_ availability [61]. Under hypoxia, cells reduce their reliance upon O_2_-dependent, mitochondrial oxidative phosphorylation but change to an O_2_-independent glycolytic pathway for the maintenance of sufficient ATP production [62].

In our study, SpO_2_ reduced, as expected, to 76.4 ± 3.8% in HYP at the end of the muscle damaging exercise protocol, whereas SpO_2_ remained at 95.5 ± 1.7% in the CON group (see Fig. 2A). These reductions occurred at a PiO_2_ of 80 mmHg in the HYP group. In line with previous research, tissue oxygenation values have been demonstrated to be lower under systemic hypoxia, including resting O_2_ oxygenation values but also VO_2max_ values [63, 64]. The reduction in SpO_2_ during exercise is in line with other studies, that evaluated capillary oxygenation under hypoxia. In moderate hypoxia (FiO_2_ of 14.4%), SpO_2_ decreased to approximately 85% during a 30 min lasting submaximal endurance performance [65]. Similarly, in the study by Nam et al. (2020), capillary oxygenation decreased after a 30 min lasting endurance task below 80% at a FiO_2_ of 12.8% [66]. Interestingly, a high-intensity interval training, performed for the duration of 30 min at a FiO_2_ of 15.2%, decreased pO_2_ to even 68% [67]. Reduced PiO_2_ levels will inevitably reduce tissue oxygenation levels [10, 68] creating an additional physiological strain that is not present during normoxic exercise [69]. In general, exercise under hypoxia has been demonstrated to increase mitochondrial density, capillary density, capillary-to-fibre ratio, fibre cross-section area, myoglobin content and oxidative enzyme activity, such as citrate synthase [70–73]. Our muscle damaging exercise protocol significantly led to symptoms of DOMS in the knee extensor muscles (Fig. 3B). Comparable subjective effects can be observed in other studies, using the same muscle damaging exercise protocol [18, 33]. Our results are also in line with another study finding, which demonstrated that DOMS significantly increased in hypoxia (FiO_2_ of 13.0%) and normoxia compared to baseline after resistance training, with no differences between groups [74]. Although DOMS is a commonly used marker for subjective ratings in this area, it has demonstrated poor correlations with changes in muscle function [75, 76]. From this perspective, measuring muscle functions like CMJ and MVIC might be of a larger interest, as especially maximum torque has been suggested to be the best measure of muscle injury, resulting from eccentric contraction [77]. In our study, both, CMJ and MVIC were reduced in both conditions (HYP and CON), with no between-group differences. After 24-hrs, MVIC values decreased to 47.4 ± 22.0% in HYP and to 56.6 ± 12.9% in CON, which can be considered as moderate muscle damage [17]. In contrast to other studies, our results did not show significant within-group changes regarding inflammatory blood markers (Table 3). Other studies demonstrated elevated higher inflammatory blood levels after using a comparable jump-protocol [34, 78, 79]. One reason for this difference might be, that our study participants were healthy females which might show a different reaction compared to males [80]. Another reason might be the large standard deviations in the normalised inflammatory blood markers (Table 3). One major problem with the assessment of pro-inflammatory markers is, that the collected values reflect not only the release into the bloodstream but also their removal [77], questioning their clinical significance in this area. However, hypoxia doesn’t seem to be the only factor of performance gains during strength training. Benavente et al. (2023) observed, that the inter-set resting intervals play a crucial role in adaptive processes. They concluded, that that loads between 60 and 80% of the one-repetition maximum that are employed ≤ 60 s show greater increases in muscle cross-sectional area, whilst the same loads with inter-set resting period of ≥120 s demonstrated greater increases in strength parameters [81].

The menstrual cycle of the healthy females might have an influence on the study outcomes. Although the oestrogen levels in both groups are comparable (Table 1), it’s possible that the exercise task might lead to different responses in females with a different hormonal status. It is reported that different oestrogen levels of females might have a protective effect on muscle damage after exercises [82]. Therefore, future studies are advised to include/assess females in a specific phase of their menstrual cycle. Additionally, the inter-individual physiological response to the hypoxic stressor should be investigated in future studies [83], especially in a female population.

Our findings support the idea, that reduce oxygen availability and tissue oxygen deprivation are the primary mechanisms for hypoxia-induced training processes. From a practical perspective, our results suggest that hypoxia can be integrated into exercise training without affecting subjective and objective recovery parameters.

## Conclusion

The results of our study show, that muscle damaging exercise under hypoxia reduces capillary- and muscle oxygenation in healthy females. Inflammatory reactions and recovery response are comparable to exercise in normoxia during a 72-hrs follow-up period.

The current results show that the effects of oxygen-restricted exercise can be incorporated into training regimes, without negatively affecting the subjective and objective recovery characteristics. These findings may facilitate practitioners in gaining a more comprehensive understanding of the impact of training under hypoxic conditions on the physiological response and recovery process.

### Electronic supplementary material

Below is the link to the electronic supplementary material.

Supplementary Material 1

Supplementary Material 2

Supplementary Material 3

## Data Availability

All data generated or analysed during this study are included in the article.

## Abbreviations

HIF-1: Hypoxia-inducible factor-1
CRP_: C-reactive protein
HYP: Hypoxia
CON: Normoxia
SpO_2_: Capillary oxygenation
SmO_2_: Muscle oxygenation
Tcore: Core temperature
Tskin: Skin temperature
ES: Exercise set
RPE: Ratings of perceived exertion
DYS: Dyspnea
DOMS: Delayed onset of muscle soreness
CMJ: Countermovement-jump
MVIC: Maximum voluntary isometric contraction
FiO_2_: Fraction of inspired oxygen
PiO_2_: Inspired oxygen tension
BSR: Blood sedimentation rate
CK: Creatine-kinase

## References

[1] Semenza GL (2009). Regulation of oxygen homeostasis by hypoxia-inducible factor 1. Physiol (Bethesda).

[2] Michiels C (2004). Physiological and pathological responses to hypoxia. Am J Pathol.

[3] West JB (1982). Man at extreme altitude. J Appl Physiol Respir Environ Exerc Physiol.

[4] Barach AL (1937). Pilot „error and oxygen want: with a description of a new oxygen face tent. JAMA.

[5] Welshman J (1998). Only connect: the history of sport, medicine and society. Int J Hist Sport.

[6] Peiser B, Reilly T (2004). Environmental factors in the summer olympics in historical perspective. J Sports Sci.

[7] Millet GP, Burtscher M, Burtscher J (2022). Is Hypoxic/Altitude training an important topic in the field of Hypoxia?. J Sci Sport Exerc.

[8] Girard O (2022). Editorial– contemporary use of Altitude Training to Reach New Heights. J Sci Sport Exerc.

[9] Wilber RL (2007). Application of altitude/hypoxic training by elite athletes. Med Sci Sports Exerc.

[10] Millet GP, Roels B, Schmitt L, Woorons X, Richalet JP (2010). Combining hypoxic methods for peak performance. Sports Med.

[11] Feriche B, García-Ramos A, Morales-Artacho AJ, Padial P (2017). Resistance Training using different hypoxic training strategies: a basis for hypertrophy and muscle Power Development. Sports Med Open.

[12] Currier BS, McLeod JC, Banfield L, Beyene J, Welton NJ, D’Souza AC (2023). Resistance training prescription for muscle strength and hypertrophy in healthy adults: a systematic review and bayesian network meta-analysis. Br J Sports Med.

[13] Silveira LS, Antunes BMM, Minari ALA, dos Santos RVT, Neto JCR, Lira FS. Macrophage polarization: implications on metabolic diseases and the role of Exercise. 2016;26(2):115–32. 10.1615/CritRevEukaryotGeneExpr.2016015920 27480774

[14] Cerqueira É, Marinho DA, Neiva HP, Lourenço O. Inflammatory effects of High and Moderate Intensity Exercise—A systematic review. Front Physiol. 2020;10. 10.3389/fphys.2019.01550 PMC6962351 31992987

[15] Eltzschig HK, Carmeliet P (2011). Hypoxia and inflammation. N Engl J Med.

[16] Kurobe K, Huang Z, Nishiwaki M, Yamamoto M, Kanehisa H, Ogita F (2015). Effects of resistance training under hypoxic conditions on muscle hypertrophy and strength. Clin Physiol Funct Imaging.

[17] Peake JM, Neubauer O, Gatta PAD, Nosaka K (2017). Muscle damage and inflammation during recovery from exercise. J Appl Physiol.

[18] Ferreira-Junior JB, Bottaro M, Vieira A, Siqueira AF, Vieira CA, Durigan JL (2015). One session of partial-body cryotherapy (-110 degrees C) improves muscle damage recovery. Scand J Med Sci Sports.

[19] Goodall S, Howatson G (2008). The effects of multiple cold water immersions on indices of muscle damage. J Sports Sci Med.

[20] Padilha CS, Figueiredo C, Minuzzi LG, Chimin P, Deminice R, Krüger K (2021). Immunometabolic responses according to physical fitness status and lifelong exercise during aging: New roads for exercise immunology. Ageing Res Rev.

[21] Khalafi M, Sakhaei MH, Symonds ME, Noori Mofrad SR, Liu Y, Korivi M (2023). Impact of Exercise in Hypoxia on Inflammatory cytokines in adults: a systematic review and Meta-analysis. Sports Med - Open.

[22] Nishimura A, Sugita M, Kato K, Fukuda A, Sudo A, Uchida A (2010). Hypoxia increases muscle hypertrophy induced by resistance training. Int J Sports Physiol Perform.

[23] Inness MW, Billaut F, Walker EJ, Petersen AC, Sweeting AJ, Aughey RJ (2016). Heavy Resistance Training in Hypoxia enhances 1RM squat performance. Front Physiol.

[24] Friedmann B, Kinscherf R, Borisch S, Richter G, Bärtsch P, Billeter R (2003). Effects of low-resistance/high-repetition strength training in hypoxia on muscle structure and gene expression. Pflugers Arch.

[25] Ho JY, Kuo TY, Liu KL, Dong XY, Tung K (2014). Combining normobaric hypoxia with short-term resistance training has no additive beneficial effect on muscular performance and body composition. J Strength Cond Res/Natl Strength Cond Assoc.

[26] Bierer BE, Meloney LG, Ahmed HR, White SA (2022). Advancing the inclusion of underrepresented women in clinical research. Cell Rep Med.

[27] Camacho-Cardenosa A, Camacho-Cardenosa M, Tomas-Carus P, Timón R, Olcina G, Burtscher M (2022). Acute physiological response to a normobaric hypoxic exposure: sex differences. Int J Biometeorol.

[28] Vento KA, Borden CK, Blacker KJ (2022). Sex comparisons in physiological and cognitive performance during hypoxic challenge. Front Physiol.

[29] Karayigit R, Eser MC, Sahin FN, Sari C, Sanchez-Gomez A, Dominguez R et al. The Acute effects of Normobaric Hypoxia on Strength, muscular endurance and cognitive function: influence of dose and sex. Biology (Basel). 2022;11(2). 10.3390/biology11020309 PMC8869765 35205175

[30] Hohenauer E, Taube W, Freitag L, Clijsen R (2022). Sex differences during a cold-stress test in normobaric and hypobaric hypoxia: a randomized controlled crossover study. Front Physiol.

[31] Moher D, Hopewell S, Schulz KF, Montori V, Gøtzsche PC, Devereaux PJ (2010). CONSORT 2010 explanation and elaboration: updated guidelines for reporting parallel group randomised trials. BMJ.

[32] Clarkson PM, Hubal MJ (2002). Exercise-induced muscle damage in humans. Am J Phys Med Rehabil.

[33] Hohenauer E, Costello JT, Deliens T, Clarys P, Stoop R, Clijsen R (2020). Partial-body cryotherapy (-135 degrees C) and cold-water immersion (10 degrees C) after muscle damage in females. Scand J Med Sci Sports.

[34] Howatson G, Goodall S, van Someren KA (2009). The influence of cold water immersions on adaptation following a single bout of damaging exercise. Eur J Appl Physiol.

[35] Kolb JC, Farran P, Norris SR, Smith D, Mester J (2004). Validation of pulse oximetry during progressive normobaric hypoxia utilizing a portable chamber. Can J Appl Physiol.

[36] Selfe J, Alexander J, Costello JT, May K, Garratt N, Atkins S (2014). The effect of three different (-135 degrees C) whole body cryotherapy exposure durations on elite rugby league players. PLoS ONE.

[37] Ryan TE, Southern WM, Reynolds MA, McCully KK (2013). A cross-validation of near-infrared spectroscopy measurements of skeletal muscle oxidative capacity with phosphorus magnetic resonance spectroscopy. J Appl Physiol (1985).

[38] Caminal P, Sola F, Gomis P, Guasch E, Perera A, Soriano N (2018). Validity of the Polar V800 monitor for measuring heart rate variability in mountain running route conditions. Eur J Appl Physiol.

[39] Byrne C, Lim CL (2007). The ingestible telemetric body core temperature sensor: a review of validity and exercise applications. Br J Sports Med.

[40] Bogerd CP, Velt KB, Annaheim S, Bongers CCWG, Eijsvogels TMH, Daanen HAM (2018). Comparison of two telemetric intestinal temperature devices with rectal temperature during exercise. Physiol Meas.

[41] Hasselberg MJ, McMahon J, Parker K (2013). The validity, reliability, and utility of the iButton(R) for measurement of body temperature circadian rhythms in sleep/wake research. Sleep Med.

[42] Borg GA (1982). Psychophysical bases of perceived exertion. Med Sci Sports Exerc.

[43] Mahler DA, Horowitz MB (1994). Perception of breathlessness during exercise in patients with respiratory disease. Med Sci Sports Exerc.

[44] Crisafulli E, Clini EM (2010). Measures of dyspnea in pulmonary rehabilitation. Multidiscip Respir Med.

[45] Tishkowski K, Gupta V. Erythrocyte sedimentation rate. StatPearls. Treasure Island (FL): StatPearls Publishing Copyright © 2022. StatPearls Publishing LLC.; 2022. 32491417

[46] Otsuji S, Shibata H, Umeda M (1982). Turbidimetric immunoassay of serum C-reactive protein. Clin Chem.

[47] Hohenauer E, Clarys P, Baeyens JP, Clijsen R. Non-invasive assessments of subjective and objective recovery characteristics following an exhaustive Jump Protocol. J Vis Exp. 2017;124. 10.3791/55612 PMC5608348 28654037

[48] Faul F, Erdfelder E, Lang A-G, Buchner A (2007). G*Power 3: a flexible statistical power analysis program for the social, behavioral, and biomedical sciences. Behav Res Methods.

[49] Cohen J (1992). A power primer. Psychol Bull.

[50] Sadiku P, Walmsley SR (2019). Hypoxia and the regulation of myeloid cell metabolic imprinting: consequences for the inflammatory response. EMBO Rep.

[51] Walmsley SR, Print C, Farahi N, Peyssonnaux C, Johnson RS, Cramer T (2005). Hypoxia-induced neutrophil survival is mediated by HIF-1alpha-dependent NF-kappaB activity. J Exp Med.

[52] Roiniotis J, Dinh H, Masendycz P, Turner A, Elsegood CL, Scholz GM (2009). Hypoxia prolongs monocyte/macrophage survival and enhanced glycolysis is associated with their maturation under aerobic conditions. J Immunol.

[53] Nissim Ben Efraim AH, Eliashar R, Levi-Schaffer F (2010). Hypoxia modulates human eosinophil function. Clin Mol Allergy.

[54] Faiss R, Girard O, Millet GP (2013). Advancing hypoxic training in team sports: from intermittent hypoxic training to repeated sprint training in hypoxia. Br J Sports Med.

[55] Govus AD, Abbiss CR, Garvican-Lewis LA, Swinkels DW, Laarakkers CM, Gore CJ (2014). Acute hypoxic exercise does not alter post-exercise iron metabolism in moderately trained endurance athletes. Eur J Appl Physiol.

[56] Sumi D, Yamaguchi K, Goto K (2021). Impact of three consecutive days of endurance training under hypoxia on muscle damage and inflammatory responses. Front Sports Act Living.

[57] Vogt M, Puntschart A, Geiser J, Zuleger C, Billeter R, Hoppeler H (2001). Molecular adaptations in human skeletal muscle to endurance training under simulated hypoxic conditions. J Appl Physiol.

[58] Zoll J, Ponsot E, Dufour S, Doutreleau S, Ventura-Clapier R, Vogt M (2006). Exercise training in normobaric hypoxia in endurance runners. III. Muscular adjustments of selected gene transcripts. J Appl Physiol.

[59] Chandel NS, Maltepe E, Goldwasser E, Mathieu CE, Simon MC, Schumacker PT (1998). Mitochondrial reactive oxygen species trigger hypoxia-induced transcription. Proc Natl Acad Sci U S A.

[60] Ameln H, Gustafsson T, Sundberg CJ, Okamoto K, Jansson E, Poellinger L (2005). Physiological activation of hypoxia inducible factor-1 in human skeletal muscle. Faseb j.

[61] Blomqvist CG, Saltin B (1983). Cardiovascular adaptations to physical training. Annu Rev Physiol.

[62] Kierans SJ, Taylor CT (2021). Regulation of glycolysis by the hypoxia-inducible factor (HIF): implications for cellular physiology. J Physiol.

[63] Richardson RS, Duteil S, Wary C, Wray DW, Hoff J, Carlier PG (2006). Human skeletal muscle intracellular oxygenation: the impact of ambient oxygen availability. J Physiol.

[64] Richardson RS, Noyszewski EA, Kendrick KF, Leigh JS, Wagner PD (1995). Myoglobin O2 desaturation during exercise. Evidence of limited O2 transport. J Clin Invest.

[65] Park HY, Kim JW, Nam SS. Metabolic, Cardiac, and hemorheological responses to Submaximal Exercise under Light and Moderate Hypobaric Hypoxia in Healthy men. Biology (Basel). 2022;11(1). 10.3390/biology11010144 PMC8772706 35053141

[66] Nam SS, Park HY (2020). Effects of endurance exercise under hypoxia on acid-base and ion balance in healthy males. Phys Act Nutr.

[67] Żebrowska A, Jastrzębski D, Sadowska-Krępa E, Sikora M, Di Giulio C (2019). Comparison of the effectiveness of high-intensity interval training in Hypoxia and Normoxia in Healthy male volunteers: a pilot study. Biomed Res Int.

[68] Vogt M, Hoppeler H (2010). Is hypoxia training good for muscles and exercise performance?. Prog Cardiovasc Dis.

[69] Buchheit M, Kuitunen S, Voss SC, Williams BK, Mendez-Villanueva A, Bourdon PC (2012). Physiological strain associated with high-intensity hypoxic intervals in highly trained young runners. J Strength Cond Res/Natl Strength Cond Assoc.

[70] Melissa L, MacDougall JD, Tarnopolsky MA, Cipriano N, Green HJ (1997). Skeletal muscle adaptations to training under normobaric hypoxic versus normoxic conditions. Med Sci Sports Exerc.

[71] Terrados N, Jansson E, Sylvén C, Kaijser L (1990). Is hypoxia a stimulus for synthesis of oxidative enzymes and myoglobin?. J Appl Physiol.

[72] Green H, MacDougall J, Tarnopolsky M, Melissa NL (1999). Downregulation of Na+-K+-ATPase pumps in skeletal muscle with training in normobaric hypoxia. J Appl Physiol.

[73] Desplanches D, Hoppeler H, Linossier MT, Denis C, Claassen H, Dormois D (1993). Effects of training in normoxia and normobaric hypoxia on human muscle ultrastructure. Pflugers Arch.

[74] Jonson AM, Girard O, Walden TP, Marston KJ, Scott BR. Hypoxia does not impair Resistance Exercise performance or amplify post-exercise fatigue. Res Q Exerc Sport. 2023:1–8. 10.1080/02701367.2023.2193232 37039734

[75] Howell JN, Chleboun G, Conatser R (1993). Muscle stiffness, strength loss, swelling and soreness following exercise-induced injury in humans. J Physiol.

[76] Rodenburg JB, de Boer RW, Schiereck P, van Echteld CJ, Bär PR (1994). Changes in phosphorus compounds and water content in skeletal muscle due to eccentric exercise. Eur J Appl Physiol Occup Physiol.

[77] Warren GL, Lowe DA, Armstrong RB (1999). Measurement tools used in the study of eccentric contraction-induced injury. Sports Med.

[78] Miyama M, Nosaka K. Muscle damage and soreness following repeated bouts of Consecutive Drop jumps. Adv Exerc Sports Physiol. 2004;10(3).

[79] Miyama M, Nosaka K (2004). Influence of surface on muscle damage and soreness induced by consecutive drop jumps. J Strength Cond Res.

[80] Schmitz B, Niehues H, Thorwesten L, Klose A, Krüger M, Brand SM (2020). Sex differences in high-intensity interval training-are HIIT protocols interchangeable between females and males?. Front Physiol.

[81] Benavente C, Schoenfeld BJ, Padial P, Feriche B (2023). Efficacy of resistance training in hypoxia on muscle hypertrophy and strength development: a systematic review with meta-analysis. Sci Rep.

[82] Williams T, Walz E, Lane AR, Pebole M, Hackney AC (2015). The effect of estrogen on muscle damage biomarkers following prolonged aerobic exercise in eumenorrheic women. Biology Sport.

[83] Soo J, Girard O, Ihsan M, Fairchild T (2020). The Use of the SpO(2) to FiO(2) ratio to individualize the hypoxic dose in Sport Science, Exercise, and Health settings. Front Physiol.

