# Supplementary material for: Acute physiological responses and muscle recovery in females: a randomised controlled trial of muscle damaging exercise in hypoxia
Source: BMC Sports Sci Med Rehabil. 2024 Mar 22;16:70. doi: 10.1186/s13102-024-00861-1 (PMC10960417; doi:10.1186/s13102-024-00861-1)
Supplement: Supplementary file 1 — Supplementary Material 1 [file 13102_2024_861_MOESM1_ESM.doc]

**Allocation**

**Analysis**

**Follow-Up**

**Enrollment**

Assessed for eligibility (n=25)

Excluded (n=0)

♦  Not meeting inclusion criteria (n=5)

Analysed (n=10)
♦ Excluded from analysis (give reasons) (n=0)

Lost to follow-up (give reasons) (n=0)

Discontinued intervention (give reasons) (n=0)

Allocated to hypoxia (n=10)

♦ Received allocated intervention (n=10)

Lost to follow-up (give reasons) (n=0)

Discontinued intervention (give reasons) (n=0)

Allocated to normoxia (n=10)

♦ Received allocated intervention (n=10)

Analysed (n=10)
♦ Excluded from analysis (give reasons) (n=0)

Randomized (n=20)
