# Supplementary material for: Acute physiological responses and muscle recovery in females: a randomised controlled trial of muscle damaging exercise in hypoxia
Source: BMC Sports Sci Med Rehabil. 2024 Mar 22;16:70. doi: 10.1186/s13102-024-00861-1 (PMC10960417; doi:10.1186/s13102-024-00861-1)
Supplement: Supplementary file 3 — Supplementary Material 3 [file 13102_2024_861_MOESM3_ESM.docx]

Supplementary materials

S1 CONSORT 2010 flowchart

S2 CONSORT 2010 checklist
